# A Longitudinal Observation Study to Investigate the Associations Between Individual Qualitative Behavioural Assessment Scores and Milk Production in Housed Dairy Cows in the UK

**DOI:** 10.3390/ani16193095

**Published:** 2026-10-01

**Authors:** Emily F. Craven, Jorge A. Vázquez-Diosdado, Jasmeet Kaler, Alison Russell, Martin J. Green, Jake S. Thompson

**Affiliations:** 1 School of Veterinary Medicine and Science, University of Nottingham, Nottingham LE12 5RD, UK; emily.craven@nottingham.ac.uk (E.F.C.);; 2 Agriculture and Horticulture Development Board (AHDB), Coventry CV3 4PE, UK

**Keywords:** QBA, positive welfare, dairy cattle, milk quality, milk production

## Abstract

Qualitative Behavioural Assessment (QBA) has been used at both the herd and individual levels as an indirect measure to assess changes in the emotional state in dairy cattle but has not been used in individual cattle to explore associations between emotional state and milk production over a lactation. The study assessed 114 individual cows over a 13-month period utilising QBA and milk production data on a single farm. QBA scores were analysed using Principal Component Analysis (PCA). A decrease in Principal Component 1 (PC1) was associated with an increase in scores for positive behavioural terms such as happy, relaxed, calm and content. An increase in Principal Component 2 (PC2) was associated with an increased score for terms such as active, playful and inquisitive. Analysis with mixed models showed that milk yield was significantly negatively associated with PC1 for first-lactation cows (estimate = −0.24, *p* = 0.016) whilst accounting for days in milk (DIM) and temporal dependence. Variations in daily milk yields, assessed using the log-transformed variance of deviations from individual lactation curves (LnVar), were significantly positively associated with PC1 (percentage change in variability = 3.63, *p* = 0.004). These results indicate that increased milk yield and reduced daily yield variation were associated with more positive behavioural QBA terms. Exploratory work using clustering of PCA scores from the first 30 DIM was associated with 100-day milk yield, with cows in the more positive clusters (cluster 2 for PC1 and cluster 2 for PC2) producing the greater yield volumes (PC1 cluster 2: estimate 529.4, *p* = 0.02; PC2 cluster 2: estimate 1158.5, *p* < 0.001). These findings suggest that cows with more positive emotion scores are associated with increased milk production and provides evidence for further exploration in this research area.

## Simple Summary

This longitudinal observational study explored whether individual Qualitative Behavioural Assessment (QBA) outcomes were associated with milk production in housed dairy cows. The results suggest that first-lactation cows receiving QBA scores consistent with more positive valence states were associated with a higher daily milk yield, and that cows with a lower milk-yield variation were also associated with more positive QBA terms. These findings are of benefit to veterinarians and farmers, highlighting that there may be a relationship between a cow’s emotional state and her productivity, indicating a need for further work exploring these relationships.

## 1. Introduction

Assessing emotional state is of fundamental importance in dairy cattle to ensure they experience positive emotions rather than just ensuring an absence of suffering [1]. Many definitions of welfare refer to affective states, a concept that is fundamental to the Five Domains Model (5DM) [2] and the emotional states part of Fraser’s model [3]. Affective states are assessed in humans with a combination of self-reporting, behavioural and physiological measures. Given physiological measures are usually invasive, and verbal self-reporting is not possible in livestock, there is an interest in developing behavioural measures as a proxy measure to infer an affective state. One such technique that has been developed is Qualitative Behavioural Assessment (QBA) [4] which aims to assume elements of an animal’s mental state [5]. This method focuses its elements on how an animal is doing an activity rather than on what activity it is performing [6]. QBA is considered a measure of positive emotional state in the Welfare Quality Assessment protocol for cattle [7]. Furthermore, behavioural monitoring, including daily eating, rumination and lying pattern assessments are increasingly being considered within data-driven approaches to dairy-cow welfare and productivity assessment [8]. This can also be combined in automatic milking systems, where productive and udder-health outcomes arise from interactions among mammary physiology, milking technology, management and cattle behaviour [9].

One of the key production metrics for dairy cows is milk yield and quality, given that it directly drives a herd’s profitability and efficiency. A herd’s aim is to optimise milk yield whilst maximising milk quality (often measured using total solids, specifically fats and proteins) and somatic cell count (SCC). Milk yield is influenced by biological and management variables and therefore whilst it cannot be considered a measure of welfare, it has been shown that milk parameters and performance are associated with welfare. Examples include Babiciu et al., 2025 [10] which showed herd-level effects and Coignard et al., 2014 [11] which found positive associations with yield and emotional state.

Furthermore, the behaviour-based Positive Affect Index, derived from QBA, was used to show that herds in Romania with more positive emotional states had lower SCCs (22% reduction, *p* = 0.016) and produced slightly more milk (+1.07 L per milking, *p* = 0.077), although this result was not statistically significant and the 95% confidence interval (CI) was −0.11–+2.25 [12]. Likewise, Craven et al., 2026 [13] showed using group-level QBA that increased temperature (0.18, *p* < 0.001), mean days in milk (0.10, *p* < 0.001), and reduced milk per cow per day (−0.67, *p* < 0.001) were significantly associated with an increase in Principal Component terms, linked to a more agitated state. Group- and individual-level factors have been shown to impact milk yield, such as diet and temperature–humidity index [14] at the group level and health factors such as mastitis [15] at an individual level. Furthermore, it has been shown that day-to-day milk-yield variation is a promising indicator for individual resilience [16], building upon work by Elgersma et al., 2018 [17] that showed that the variance of the daily recorded milk yield was heritable and that the variance of milk production performed best among the fluctuation traits tested to predict udder health, ketosis, and longevity.

At an individual level, it has been shown that QBA can be used to investigate emotional expression in dairy cows affected by an experimental intramammary challenge (mastitis) with *Escherichia coli* [18]. Furthermore, Judgement and Attention Bias Tasks have been used to show that milk-derived indicators were affected by the personality traits of activity, fearfulness and sociability [19]. Hedlund et al., 2015 [20] showed a relationship between behavioural responses during milking (stepping and kicking behaviour) and in personality tests (responses to novel object and social isolation) and milk production, where cows showing signs of nervousness produced less milk. A meta-analysis and systemic review using nine studies found that most of the publications analysed reported a negative association between reactive temperament and milk yield, indicating that calmer cows would produce more milk. However, the study concluded that the results did not confirm the hypothesis that calmer cows would be the most productive for both daily and total yield [21]. Furthermore, recent evidence from Holstein heifers indicated that individual coping styles are associated with behavioural responses during automatic milking and habituation to the robotic milking environment [22]. By using techniques such as flight speed, and judgement bias, these studies explore related concepts in terms of temperament, personality and affective state, but do not necessarily represent the same construct as QBA.

QBA has been used in individual cows to demonstrate the variation in emotional state in dairy cows during early lactation [23] but the longer-term effects and production effects have not been explored. This study aimed to use prospective QBA of individual dairy cows to investigate associations between behavioural scores and milk production, both in terms of milk yield and milk quality. Given the studies seen using different constructs, it was hypothesised that more positive behavioural scores would be associated with higher milk yield, lower yield variability and better milk quality (defined as higher milk solids and lower SCC). Exploratory analysis was also proposed for early-lactation clustering analysis.

## 2. Materials and Methods

Ethical approval was granted by the University of Nottingham School of Veterinary Medicine and Science Committee, number 3917-230824. All cow-based measures were observational with no direct interventions on the cattle undertaken.

### 2.1. Animals and Housing

The study was performed on a dairy farm in north-east Derbyshire between 3 June 2024 and 7 July 2025 as described in [13]. The herd consisted of 270 pedigree Holstein cows in an all-year-round housed automatic milking system (AMS) (31.3 kg/cow/day, previous 12-month average at time of data completion). The robots were GEA DairyRobot R9500 (GEA Group, Dusseldorf, Germany) with three robots per group (stocking rate of approximately 45 cows per robot). Housing for lactating cows consisted of two equal-sized groups (approximately 69 m × 15.5 m) in a sawdust-based mattress cubicle system (284 cubicles in a three row design). The cows had access to concentrate during milking and partial mixed ration (PMR) comprising a maize and grass silage-based mix with a mineral blend at the feed-face. PMR was provided in the morning at 08:00 h and regularly pushed up in the day using a robot (GEA DairyFeed F4800, GEA Group, Dusseldorf, Germany) to provide an effectively ad libitum system. Slurry management was performed using automatic scrapers.

### 2.2. Sample Size

Cattle were recruited to the trial based upon the herd’s death and exit rate as the study aimed to have over 100 cows complete the trial at approximately 250 days in milk. An a priori power calculation was undertaken based on the sensitivity analysis from repeat stochastic simulation outcomes of mixed-effects models and determined that 100 cows with 25 repeated measurements per cow was appropriately powered to detect a 1 L difference in daily milk yield associated with a 0.2-unit difference in a Principal Component term, with a significance level of 0.05. Data was simulated assuming a mean milk yield of 28 kg/day (SD = 6), a between-cow random-effect SD of 1.5, and a residual SD of 1.2. Three repeated observations were simulated per cow, and the model included cow as a random intercept. Sample sizes of 10, 20, 30, 40, 50, 75, 100, and 150 cows with 500 simulated datasets were analysed using linear mixed-effects models. A sensitivity analysis was additionally performed by varying the assumed between-cow SD from 0.8 to 2.5.

### 2.3. Recruitment

Multi and primiparous cows were recruited at the visit closest to the point of calving meaning that cows were recruited at 0–3 days in milk (recruitment visits took place on Monday, Wednesday, Friday). All cows which calved were enrolled with no additional criteria for exclusion applied. A total of 25 in lactation observations per cow were planned.

The assessment frequency was:

0–14 days on trial: 3 × week (M, W, F) total 6 observations;

14–28 days on trial: 2 × week (M, F) total 4 observations;

28–63 days on trial: weekly (M) total 5 observations;

63–105 days on trial: fortnightly (M) total 3 observations;

105–245 days on trial: monthly (M) total 7 observations but with 3 observations at week 34/35 (M, F, M or F, M, F).

### 2.4. QBA

As in Craven et al., 2026 [13], one trained assessor (EFC) observed the cows and performed the QBAs up to three times per week. The Welfare Quality (WQ) assessment protocol for dairy cattle [7] was followed, whereby 20 descriptors were scored: active, relaxed, fearful agitated, calm, content, indifferent, frustrated, friendly, bored, playful, positively occupied, lively, inquisitive, irritable, uneasy, sociable, apathetic, happy, and distressed. Cows were observed from the office which is a raised platform with large windows on all aspects, also serving as a viewing area [13]. Binoculars were used to ensure that the observer had a clear view of subtle expressions in all areas of the shed. If cattle could not be identified from the raised office, then they were observed at the cow level, but time was allowed for the cows to habituate if the observer had been spotted. Both methods have been described in the literature; for example Russell et al., 2023 [24] used a raised platform and Ferguson et al., 2025 [25] performed the QBAs amongst the cattle. Being spotted was defined as the cows changing their behaviour or showing signs of interaction with the observer. Habituation was defined as the cow returning to her previous behaviour and ceasing any interaction with the observer. Observations were performed at 10:20 h following a whole-group observation. This was chosen to give the best indication of the herd’s undisturbed behaviour based upon the farm’s routine and to ensure that the cattle were routinely free from any form of human disturbance. For further information see Craven et al., 2026 [13].

The individual cows were observed for approximately 2 min in total. There is not a currently recognised standard observation time, or generally accepted protocol for performing individual QBAs in cattle. However, 2 min is a compromise between previously published protocols, ranging from approximately 30 s [26] to 5 min [18], as well as being the time used in Craven et al., 2026 [23].

To achieve consistency and try to observe each cow at a similar time point each day, the shed was assessed systematically. Observations always started at the far-left hand corner and then the observer scanned down the cubicles, back up the feed-face, before finding any animals that were missed on that side. Once the left group was finished then the same procedure was performed on the right group. Any cattle unable to be located from the office were assessed at the cow level. The shed was assessed in the same direction in time to try to ensure consistency in time recording as time may cause variation in QBA. Cows were not scored when they were being milked as their body language could not be suitability observed as they were mostly obscured when in the robot cell and so were later found and scored post milking. Cattle tended to be freely distributed amongst the shed, apart from on very hot days where they tended to gather in the bottom corners. This may have created systematic associations between cow location and observation time, but given QBA measures how the behaviour is performed, rather than what the behaviour being performed was, this should not have affected the data.

### 2.5. Scoring

Immediately following an observation, the observer turned away from the individual cow being assessed to perform the scoring, ensuring that the scoring was based solely on the observation period. This was done using a paper copy of the WQ QBA template [7], whereby the 20 individual descriptive terms were scored by placing a mark on the 125 mm Visual Analogue Scale (VAS). For each descriptive term, the left side on the scale (0 mm) represents the minimum expression and the right side (125 mm) represents the maximum expression. Given this, the descriptive terms (listed in Section 2.4) are both positive and negative: for positive terms, such as happy, the expression is more positive as the number increases; for negative terms, such as fearful, the expression is more negative [13].

Following the completion of the observations, each line point was manually measured in mm from the minimum point on the scale to the assessment mark, giving a value for each descriptive term between 0 and 125 mm. These were measured to the nearest mm using a standard 30 cm ruler and inputted into a spreadsheet for further analysis (see QBA analysis). All data was checked during inputting.

### 2.6. Production Data

Production data (daily yields) were taken from the farm management software (DairyNet, GEA, Dusseldorf, Germany). The milk production data was collected directly by the milking robots at each milking event. Somatic cell count (SCC) and milk solids were derived from monthly milk recording data (CIS- The Cattle Information Service, Shropshire, UK) taken from each individual cow monthly as part of routine management.

### 2.7. Statistical Analyses

The data was analysed in Microsoft Office Excel and RStudio version 4.4.1 [27]. Additional packages used in RStudio were *ggcorrplot* [28], *factoextra* [29,30], *ggplot2* [30], *dplyr* [31], *tidyverse* [32], *psych* (W Revelle, 2022), *merTools* (Knowles and Frederick (2025), *performance* [33], *Lmtest* [34], *DHARMa* [35], *Splines* [36], *influence.ME* [37], *forecast* [38], *lme4* [39], and *boot* [40].

### 2.8. Intra-Observer Reliability

Intra-observer reliability was tested using Intraclass Correlation Coefficient (ICC) (3,1) for PC1 and PC2. ICC was chosen to compare absolute agreement rather than associations. The same ten training videos were observed three times (beginning, middle and end of the trial). The ICC for both was 0.9 or above, indicating high reliability (PC1 0.98, 95% CI 0.94–0.99, *p* < 0.001; PC2 0.90, 95% CI 0.73–0.97, *p* < 0.001). Further information is given in [13].

### 2.9. QBA Analysis (PCA)

The measurements were normalised using the scale function (achieved by dividing the (centred) columns of the descriptor terms by their standard deviation), before being further analysed using a Principal Component Analysis (PCA) [41]. Descriptive assessment of QBA was conducted to facilitate visualisation of the important term variables contributing to the key principal components.

Given QBA assessments were repeated longitudinally within cows, individual assessments were not considered statistically independent. Confidence intervals for PCA loadings were therefore established using a cluster bootstrap with cow as the resampling unit. For each of the 2000 bootstrap replicates, 114 cows were sampled with replacement, and all available QBA observations for each selected cow were retained. The PCA was repeated for each bootstrap sample, and PC1 and PC2 were matched to the corresponding components of the original PCA and their signs aligned. Ninety-five percent confidence intervals of the bootstrap loading distributions and loadings were considered stable if their confidence intervals did not include zero.

### 2.10. Model 1: Is Daily Milk-Yield Production Associated with Principal Component Score?

Total daily milk yield was modelled as the outcome variable using a linear mixed-effects model with Gaussian errors. PC1, PC2, and lactation number (categorical: lactation 1, 2, and 3+) and their interactions were modelled as linear fixed effects whilst DIM was modelled using natural cubic splines to account for the non-linear biological relationship between milk yield and stage of lactation. Lactation number was included to account for parity-related differences. Cow ID and date were included as random effects to account for repeated measures, shared environmental effects and individual animal variation. Incorporating non-linear effects substantially improved model fit compared with linear models with model selection based on likelihood ratio tests and Akaike’s Information Criterion (AIC). Additionally, to account for repeated observations within cows and the fact that they were collected at irregular time intervals, a continuous Ornstein–Uhlenbeck (OU) correlation structure was included to account for temporal autocorrelation. Inclusion of the OU correlation structure substantially improved model fit compared with a model containing only cow and date random effects.

The model was fitted using the *glmmTMB* package in R [42] and can be expressed as

$$y_{ij}=\beta_{0}+\beta_{1}{\mathrm{PC}1}_{\mathrm{ij}}+\beta_{2}{\mathrm{PC}2}_{\mathrm{ij}}+\beta_{3}{\mathrm{Lactation}}_{\mathrm{ij}}+\beta_{4}{\left(\mathrm{PC}1\;\times \;\mathrm{Lactation}\right)}_{\mathrm{ij}}\;+\beta_{5}{\left(\mathrm{PC}2\;\times \;\mathrm{Lactation}\right)}_{\mathrm{ij}}+f{\left(\mathrm{DIM}\right)}_{\mathrm{ij}}+b_{i}+c_{j}+\epsilon_{ij}$$

where $y_{ij}$ represents total milk yield for cow *i* on date *j*, $\beta_{0}$ is the intercept, β terms represent fixed-effect coefficients, PC1 and PC2 are the principal component scores, f(DIM) denotes a natural cubic spline for days in milk with five degrees of freedom, $b_{i}$ represents the random intercept for cow, $c_{j}$ represents the random intercept for date, and $\epsilon_{ij}$ is the residual error term. Residuals within cows were modelled using a continuous-time OU correlation structure.

Ninety-five percent confidence intervals for fixed-effect parameter estimates were calculated using profile likelihood methods from the fitted linear mixed-effects model.

Model assumptions were assessed using simulated residual diagnostics and examination of within-cow residual correlations as a function of time between observations.

Where interactions between PC scores and lactation were included, estimated slopes of each PC within each lactation were obtained using the emmmeans package [43]. Pairwise comparisons of slopes between lactations were adjusted using Tukey’s method.

### 2.11. Model 2: Are Variations in Daily Milk Yields Associated with Principal Component Score?

To assess whether day-to-day deviations in milk production were associated with principal component score, within-cow individual milk-yield deviations were calculated from a predicted value based on whole lactation data. Daily milk-yield records were aggregated at the cow and day level using the sum of all milkings in a 24 h period. Only cows with more than 300 daily milk recordings were retained for analysis to ensure the curves were based on sufficient longitudinal milk-yield information. Days in milk (DIM) between 0 and 305 were included.

Individual lactation trajectories were estimated separately for each cow using quantile regression at the 70th percentile (τ=0.7) with a fourth-order polynomial function of DIM using the quantreg package [44] and the poly function in R. The variance was log-transformed to make it more normally distributed [17]. Quantile regression was used to reduce sensitivity to transient low-yield observations while characterising the expected lactation curve. The 0.7 quantile was used based upon Poppe et al., 2020 [16] because a quantile regression curve using a quantile greater than 0.5 was expected to better match the potential milk yield in absence of disturbances and therefore was expected to generate deviations that contain more information on resilience.

The estimated regression coefficients for each cow were then used to calculate individual expected lactation curves. Low variance of the deviations is thought to indicate good resilience [16], because resilient cows have a smaller range of deviations from their lactation curve than less-resilient cows do, so the effect on principal component was tested so see if affective state is associated with resilience. The first and last 10 DIM of each cow were excluded from calculation of the resilience indicators [16]. For further information on log-transformed variance of deviations from individual lactation curves calculations (LnVar) see Poppe et al., 2020; Elgersma et al., 2018 [16,17].

The fitted lactation model for each cow was

$$Q_{0.7}(Milk_{it})=\beta_{0}+\beta_{1}DIM_{it}+\beta_{2}DIM_{it}^{2}+\beta_{3}DIM_{it}^{3}+\beta_{4}DIM_{it}^{4}$$

where $Milk_{it}$ represents daily milk yield for cow i on day t.

Residual milk-yield deviations were calculated as

$$e_{it}=Milk_{it}-{\hat{Milk}}_{it}$$

Short-term variability in milk yield was quantified using a 7-day rolling variance of residuals within each cow:

$$Var_{it}=Var(e_{i,t-6},\ldots ,e_{it})$$

The rolling variance was log-transformed to improve normality and stabilise variance:

$$LnVar_{it}=log(Var_{it})$$

Associations between behavioural principal components and milk-yield variability were evaluated using a linear mixed-effects model, including PC1, PC2 and lactation number as fixed effects, with random intercepts for Cow ID and date to account for repeated observations within cows and shared day-level effects.

The model was specified as

$$LnVar_{it}=\beta_{0}+\beta_{1}PC1_{it}+\beta_{2}PC2_{it}+\beta_{3}Lactation_{it}+u_{i}+v_{t}+\varepsilon_{it}$$

where $LnVar_{it}$ represents the natural logarithm of the rolling variance for observation t from cow i, $\beta_{0}$ is the intercept, and $\beta_{1}$–$\beta_{3}$ represent fixed-effect regression coefficients for PC1, PC2, and lactation number, respectively. The term $u_{i}$ represents the random intercept effect of cow, assumed to follow a normal distribution $u_{i}∼N(0,\sigma_{Cow}^{2})$, while $v_{t}$ represents the random intercept effect of date, assumed to follow $v_{t}∼N(0,\sigma_{Date}^{2})$. The residual error term was denoted by $\varepsilon_{it}∼N(0,\sigma^{2})$.

As milk-yield variability was natural-log-transformed (LnVar), model coefficients were back-transformed to the original scale and expressed as percentage changes in milk-yield variability for biological interpretation. Ninety-five percent confidence intervals for fixed-effect parameter estimates were calculated using profile likelihood methods from the fitted linear mixed-effects model.

Model assumptions were assessed through inspection of residual plots to evaluate normality and homoscedasticity. Marginal and conditional R^2^ values were calculated to quantify variance explained by fixed effects alone and by the full mixed model, respectively.

The structure of the LnVar calculation meant that observations from the same cow at nearby DIM were expected to be temporally correlated. Given that the QBA observations were not equally spaced in DIM due to the sampling structure, temporal autocorrelation was accounted for using a continuous-time first-order autoregressive [CAR(1)] correlation structure to allow the correlation between observations to decrease as the interval between DIM increased. The sensitivity model included PC1, PC2 and lactation as fixed effects and a random intercept for Cow ID, with the CAR(1) correlation structure specified within Cow ID, using DIM as the time variable. The resulting estimates and statistical significance were compared with those from the primary model to assess whether accounting for temporal autocorrelation altered the conclusions and the simplest model was retained.

### 2.12. Model 3: Is 100-Day Milk Yield Associated with First 10 Score Principal Component Cluster?

Cattle were clustered according to their behavioural patterns based upon PC1 and PC2 during the first 10 samples (approximation of first 30 days in milk). The first 30 days were used as it has previously been shown that clustering in early lactation detects different behavioural patterns [23] and 30 days were used to reflect the transition period. The mcclust package was used to process an MCMC sample of clusterings [45] to find a single best clustering to represent the sample, which are based on the posterior similarity matrix or a relabelling algorithm [23,46]. Cows were allocated to a cluster based upon the maximum posterior probability [47]. Models containing between one and ten clusters were fitted to the data and the optimal clustering solution was selected using the model-selection procedure based on the Bayesian Information Criterion (BIC) implemented by mclust.

Cluster-specific mean values were extracted to characterise the patterns for both PC1 and PC2 across the observed QBA scores (1–10). These mean profiles were visualised using line plots, allowing comparison of the response patterns between the identified clusters.

The association between-cluster membership derived from Principal Component Analysis (PCA) and 100-day milk yield was evaluated using a linear regression model. Cluster membership was included as a categorical explanatory variable with the first cluster used as the reference category. The model was fitted for clusters derived from the first and second principal component structures.

The model was specified as

$$Y_{i}=\beta_{0}+\beta_{1}X_{1i}+\beta_{2}X_{2i}+\varepsilon_{i}$$

where $Y_{i}$ represents 100-day milk yield for animal i, $X_{1i}$ represents the PC1 cluster classification, $X_{2i}$ represents the PC2 classification, $\beta_{0}$ is the intercept, $\beta_{1}$ and $\beta_{2}$ are regression coefficients, and $\varepsilon_{i}$ is the residual error term.

### 2.13. Model 4: Is Milk Quality Associated with Principal Component Scores?

Milk recording data was linked to QBA scores where it was taken within 24 h of a milk recording using fuzzyjoin [48] (either 24 h before, the same day or 24 h afterwards). Linear mixed models were then used to assess for relationships with milk constituents and somatic cell counts and principal component score. Fixed effects included behavioural principal components (PC1 and PC2), lactation number, and days in milk (DIM). Random intercepts for Cow ID were included to account for repeated observations within cows.

The model was specified as

$${Solid}_{it}=\beta_{0}+\beta_{1}PC1_{it}+\beta_{2}PC2_{it}+\beta_{3}Lactation_{it}+\beta_{4}DIM_{it}+\beta_{5}{Yield}_{it}+u_{i}+\varepsilon_{it}$$

where ${Solid}_{it}$ represents the butterfat value for observation t from cow i, $\beta_{0}$ is the intercept, and $\beta_{1}$–$\beta_{5}$ represent fixed-effect regression coefficients for PC1, PC2, lactation number, DIM and daily yield, respectively. The term $u_{i}$ represents the random intercept effect of cow, assumed to follow a normal distribution $u_{i}∼N(0,\sigma_{Cow}^{2})$. Residual errors were assumed to be normally distributed such that $\varepsilon_{it}∼N(0,\sigma^{2})$.

Model assumptions were assessed through inspection of residual plots to evaluate normality and homoscedasticity. Marginal and conditional R^2^ values were calculated to quantify the variance explained by fixed effects alone and by the full mixed model, respectively.

Somatic cell count was converted to a binary outcome (0 = below threshold, 1 = above threshold). The threshold was less than or greater than/equal to 200,000 cells/ml as this is considered the threshold of whether is likely to be affected by clinical or subclinical mastitis [49].

The probability of elevated SCC was analysed using a logistic regression model fitted in R using a generalised linear model with a binomial error distribution and logit link function. The binary response variable indicated whether somatic cell count exceeded the predefined threshold (200,000 cells/mL). Fixed effects included the first two principal component scores (PC1 and PC2), the date differential between milk recording and QBA, lactation number, and days in milk (DIM). The model was specified as

$$\mathrm{logit}(p_{i})=\beta_{0}+\beta_{1}{\mathrm{PC}1}_{i}+\beta_{2}{\mathrm{PC}2}_{i}+\beta_{3}{\mathrm{DateDiff}}_{i}+\beta_{4}{\mathrm{Lactation}}_{i}+\beta_{5}{\mathrm{DIM}}_{i}$$

where $p_{i}$ represents the probability that observation i had elevated somatic cell count, $\beta_{0}$ is the intercept, and $\beta_{1}$–$\beta_{5}$ represent regression coefficients for PC1, PC2, date differential, lactation number, and days in milk, respectively.

Some cows contributed more than one SCC observation, so observations were clustered by cow with standard errors adjusted for clustering at the cow level to account for potential-within-cow correlation. A binomial GLMM with cow as a random intercept was also considered; however, the model exhibited convergence and identifiability issues, with a very large estimated random-effect variance and was therefore not used for inference.

## 3. Results

A total of 114 cows were recruited to the trial and 102 completed the full observation period, with 12 exits. Exits were for health or production reasons. Detailed reasons for exits were not available. Lactation number was treated as a categorical variable: there were 38× parity one, 41× parity two and 35× parity 3+ cows). For all the cows recruited, mean days in milk at recruitment was 1.17 days (median 1, range 0–3, IQR 1–2), and at exit was 229.27 days (range 3–252 days) with a mean number of QBA scores of 23.9 (median 25, range 1–25). Mean daily yield was 30.3 kg (IQR 21.6–37.9 kg).

### 3.1. PCA

Principal Component Analysis of the QBA scores identified five principal components with eigen values > 1 and additional parallel analysis suggested retention of four components. Examination of the loading patterns showed that PC1 was characterised by valence-type descriptors and was therefore interpreted as valence. PC2 was characterised predominantly by activity- and arousal-type descriptors and was interpreted as arousal/activity. PC3 showed a pattern primarily involving indifference, boredom and apathy versus activity-related descriptors, while PC4 showed a less coherent pattern involving frustration, distress and sociability. As PC3 and PC4 did not clearly represent additional broad dimensions as outlined by the dimensional framework, specified as valence and arousal based upon the circumplex core affect model, first described by Russell 1980 [50] and subsequently adapted by Mendl et al., 2010 [51]. The first two principal components (PC1 and PC2) which explained the highest percentage of the variation and with eigen values > 1.0 were retained for additional inferential analysis based upon a compromise between statistical and theoretical considerations.

The first two principal components (PC1 and PC2) explained 46.1% of the variance in the data with PC1 contributing 36.4%, with 19 stable loadings, and PC2 contributing 9.7% with 17 stable loadings. Although PC3 and 4 explained 7.9 and 6.0% of the variation they did not aid biological interpretation.

PC1 was negatively associated with positively occupied, relaxed, calm, content and happy and positively associated with terms such as fearful, uneasy and apathetic, suggesting that a decrease in PC1 showed a more positive emotional state. PC2 contributed 9.7% of the variance and was positively associated with lively, playful, inquisitive and active, suggesting an increase in PC2 was associated with a more active state. Table 1 displays the relationship between the variable and the stable loadings with their associated 95% confidence intervals. For PC1, 19 factors were included as stable loadings. Inquisitive was excluded as not being a stable loading. For PC2, 17 factors were included as stable loadings. The three excluded terms were relaxed, indifferent and apathetic.

### 3.2. Model 1: Is Daily Milk-Yield Production Associated with Principal Component Score?

Inclusion of the OU temporal correlation structure substantially improved model fit compared with the corresponding model without temporal correlation (AIC= 17,501.0 compared to 18,220.7, likelihood-ration χ^2^ = 723.7, df = 2, *p* < 0.001). In the model without temporal correlation, within-cow residual correlations were strongest for observations collected within 2 days (*r* = 0.49) and declined with increasing time separation. Following inclusion of the OU structure, residual correlations were close to zero across time intervals, indicating that temporal dependence was adequately accounted for.

The final model (Table 2A) revealed significant negative effects of PC1 (estimate = −0.241, SE = 0.100, *p* = 0.016) indicative of an improvement in emotional state (such as happy, calm, content and relaxed) with increased milk-yield production for lactation 1 (reference category). Due to their known impacts on production, lactation number and DIM were also accounted for. Lactation 2 and 3+ cows gave more milk per day than lactation 1 (7.35, *p* < 0.001 and 1.06, *p* = 0.25, respectively), but the yield-related effects as reported for PC1 were less pronounced. There was evidence of an interaction between PC1 and lactation number (PC1 and lactation 3+: estimate = 0.388, SE = 0.139, *p* = 0.005). Estimated PC1 slopes indicated a negative association between PC1 and total milk yield in lactation 1 cows (estimate = −0.241, SE = 0.100, *p* = 0.016) whereas the associations were not significant in lactation 2 (estimate = −0.047, SE = 0.03, *p* = 0.610) or lactation 3+ cows (estimate = 0.147, SE = 0.103, *p* = 0.152) (Table 2B). The PC1 slope differed between first and third lactation cows (difference = −0.388, SE = 0.139, Tukey- adjusted *p* = 0.014), but not between first and second lactation cows (*p* = 0.302) or second and third lactation cows (*p* = 0.310) (Table 2C).

There was no evidence of an interaction between PC2 and lactation as shown in Table 2A–C.

Simulated residual diagnostics indicated no substantial deviations from the assumed error distribution, dispersion or outlier structure.

### 3.3. Model 2: Are Variations in Daily Milk Yields Associated with Principal Component Score?

Daily variation (LnVar) was assessed to investigate whether day-to-day deviations in milk production were associated with principal component score for each individual cow (Table 3A). As milk-yield variability was natural-log-transformed (LnVar), model coefficients were back-transformed to the original scale and expressed as percentage changes in milk-yield variability. LnVar was strongly positively associated with PC1, suggesting an increase in milk-yield variation was associated with more negative QBA terms (estimate = 0.036, SE = 0.012, *p* = 0.004, corresponding to a 3.63% change in variability). Lactation 2 also showed a significant positive effect relative to the reference (lactation 1) (estimate = 0.327, SE = 0.119, *p* = 0.007, corresponding to a 38.66% change in variability). PC2 and lactation 3+ were not statistically significant.

Random-effect variance estimates were 0.186 for Cow ID and 0.008 for date, with a residual variance of 0.664, suggesting that most unexplained variability remained at the observation level.

Residual diagnostics indicated approximately symmetric residuals, although some outliers were present (scaled residual range: −4.92 to 4.31).

Model assumptions were evaluated using residual-versus-fitted and normal QQ plots. Residuals were approximately normally distributed with minor deviations in the tails, indicating several moderate outliers. Residuals were randomly scattered around zero with no substantial evidence of heteroscedasticity or nonlinearity, suggesting that model assumptions were adequately satisfied. Accounting for within-cow temporal autocorrelation did not materially change the estimated associations or the conclusions of the primary analysis but is included in Table 3B for reference.

### 3.4. Model 3: Is 100-Day Milk Yield Associated with First 10 Score Principal Component Cluster?

**Figure 1:**
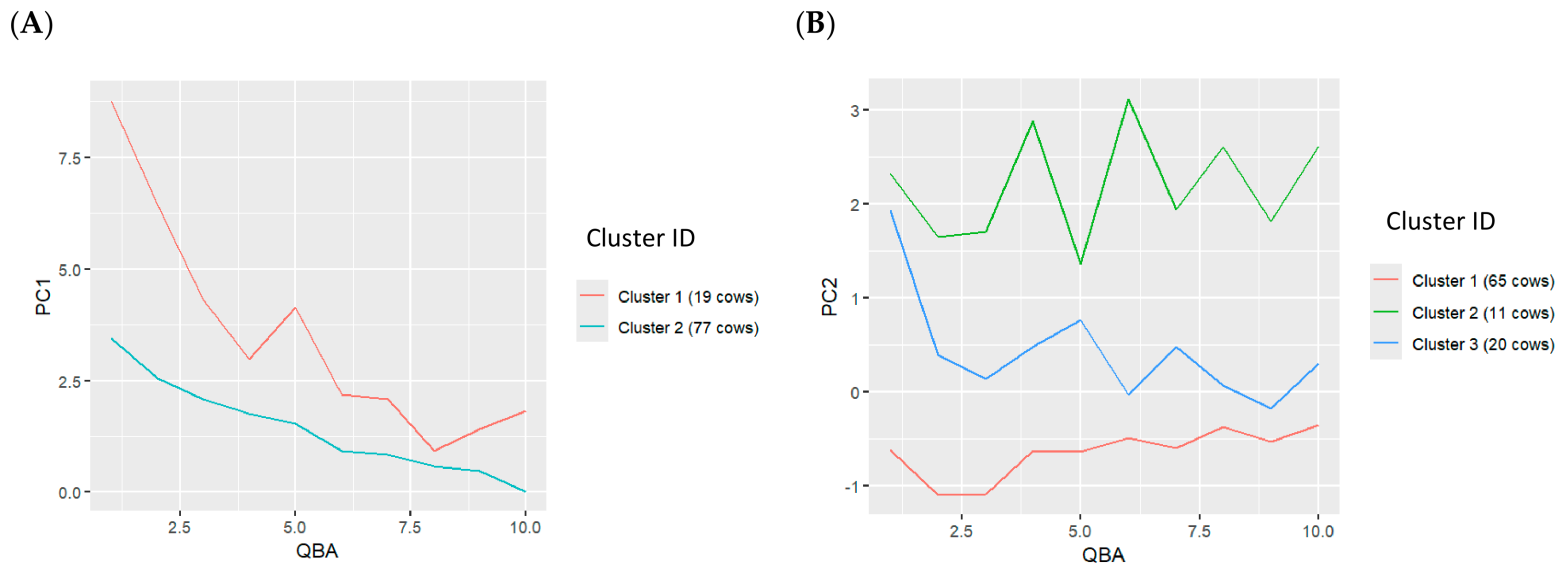
Cluster data representing the behavioural profiles of the cows for the first 10 QBA scores (first month in milk). The principal component score shown represents the mean PC score shown for cows allocated to the cluster on the y axis and QBA represents the observation number on the x axis. (**A**) PC1 split into two clusters with cluster 1 (19 cows) showing significantly worse valence (inferred from the PC score profiles) but a more marked improvement and 77 showing an improvement in valence but to a lesser extent. (**B**) PC2 split into 3 clusters with the majority in cluster 1 characterised by a relatively steady state with V2 (11) having the most variation and highest PC2 scores. In total, 96 cows were included in the final analysis as complete data was required for the first 10 QBA scores.

Exploratory data showing the profile patterns when cattle were clustered into their behaviour groups according to their principal component scores for the first 10 scores (4 weeks) are shown in Figure 1. In Figure 1A, PC1 split into two clusters with 19 cows showing significantly worse valence (cluster 1) but a more marked improvement and 77 cows showing an improvement in emotional state but to a lesser extent (cluster 2). In Figure 1B, PC2 split into three clusters with the majority in cluster 1 (65) showing a relatively steady state with cluster 2 (11) having the most variation and highest PC2 scores suggestive of a more active state. The cluster 3 group (20 cows) showed a rapid decrease in PC2 in the first two QBA scores and then remained at steady state, suggesting the cows were ‘bridging the gap’ between cluster 1 and cluster 2.

The cows in cluster 2 for PC1 (the steadier state, ‘happier’ cows) yielded significantly more milk (529.4 L for 100-day yield, SE 225.8, *p* = 0.02) (Table 4). Likewise, cluster 2 cows for PC2 (the 11 cows with the highest PC2 scores) yielded significantly more (1158.5 L for 100-day yield, SE 282.0, *p* < 0.001) and cluster 3 (20 ‘bridging the gap’ cows) yielded more than cluster 1 but less than cluster 2 (552.6 L for 100 days, SE = 219.8, *p* = 0.01). Given a higher PC2 was associated with more positive emotional traits and a lower PC1 score, this suggests that identifying the ‘happier/more active’ cows in the first month may be associated with milk yield, particularly for PC1 and PC2 at 100 days. The multiple R^2^ was 0.186 and adjusted R^2^ was 0.158.

Residual diagnostics indicated approximately symmetric residuals, although some outliers were present (scaled residual range: −1795.24 to 1921.50). Model assumptions were evaluated using normal QQ plots. Residuals were approximately normally distributed suggesting that model assumptions were adequately satisfied.

### 3.5. Model 4: Is Milk Quality Associated with Principal Component Scores?

Significant associations between QBA score were observed for total butterfat but not total protein, although the low number of observations mean that the results are considered to be exploratory. The results of the total butterfat are given in Table 5 and show that higher fat and protein were associated with a more negative PC1 (Fat: PC1 estimate −0.027, SE = 0.010, *p* = 0.008), a theme that was associated with a more positive affective valence. For fat, marginal R^2^ was 0.957; conditional R^2^ was 0.681. Random-effect variance estimates were 0.069 for Cow ID, with a residual variance of 0.012, suggesting that the variability was at the cow level.

Residual diagnostics indicated approximately symmetric residuals, although some outliers were present (fat: scaled residual range: 2.00 to 1.82).

Model assumptions for both were evaluated using residual-versus-fitted and normal QQ plots. Residuals were approximately normally distributed in the central region with deviations in the tails. Residuals were generally centred around zero with no substantial evidence of heteroscedasticity. A slight positive trend was visible suggesting that the model may underpredict at higher fitted values and overpredict at lower fitted values. No statistically significant associations for protein were seen when accounting for yield.

PC1 was positively associated with somatic cell count (SCC) of >200,000 (estimate = 0.511, SE = 0.204, *p* = 0.012) suggesting worse emotional state was associated with raised cell counts (Table 6) and PC1 was positively associated with the odds of having an SCC > 200,000 cells/mL. The differential as to when the QBA was performed or lactation number was not statistically significant, possibly due to the small sample size (90 measurements, 74 individual cows and 19 SCC positive results). Odds ratios were calculated to improve biological interpretability (such as for PC1: OR = e^0.511^ = 1.67) and are in Table 6.

The model demonstrated moderate discriminatory ability, with an area under the receiver operating characteristic curve (AUC) of 0.75 (95% CI: 0.62–0.87). The Brier score was 0.145 indicating the overall accuracy of the predicted probabilities and there was no evidence of problematic multicollinearity with all adjusted GVIF values below 2.0.

## 4. Discussion

This study followed 114 cows over their lactation (either to approximately 250 days in milk, or until herd exit, whichever was sooner) using QBA and milk performance data (both in terms of milk volume and milk quality) to see if associations could be identified. The findings suggest that changes in daily milk production are associated with behavioural changes (as shown through PC1 and PC2) from individual animal QBA scores, which provides evidence for a possible link with individual cow affective state outcomes. Although this study is novel, in that QBA has not been used on individual cattle to assess associations with milk production longitudinally over extended time periods, the results are largely in agreement with equivalent research in the literature and expand on concepts discussed.

Model one suggested that more positive (e.g., happy, relaxed, calm and content) and more active behaviours are associated with milk yield, offering a potential link between increased yield and more positive emotional states in first-lactation cows. Although at an individual level, these findings agree with the previous literature performed at the group level, these were performed using variations on welfare assessment. For example, although Coignard et al., 2014 [11] suggested that milk yield should not be used as an indicator of overall welfare, they did show that positive associations were identified between milk yield and good emotional state of the herd using Welfare Quality. It is interesting that the lactation 3+ cows did not have a significantly higher yield than parity one cows, as parity three cows would have been expected to yield significantly more. It is likely that this result was affected by older cows that were included in this group which yielded less and the trends affected the group means.

The associations between individual-level welfare assessment metrics and within-herd differences in daily milk yield should be explored further to establish the causality relationships present between these outcomes. This is particularly relevant because production differences within AMS herds may also reflect feeding and grouping strategies as shown by recent commercial-farm evidence which indicates that the management of cows according to production level can be associated with differences in feed efficiency and productive performance [52]. A potential impactful outcome from this work is to explore how within-herd yield variation could be part of the consideration as a welfare indicator. As further description into this, yield performance within a group could provide insights into the proportion of cows that are being impacted by the environment and management they are exposed to. As a hypothesis from this study that needs further testing is that higher yielding cows relative to the group may maintain positive emotions more readily compared to their counterparts which are lower in the group hierarchy. Beyond social rank and competition, QBA, milk yield, milk variability and health may all be influenced by common underlying conditions and factors such as body condition score, feed intake, ration changes, and environmental changes.

Variability in milk yield at an individual level has been suggested to be linked to resilience. At an individual level, Kremer et al., 2026 [19] described how personality factors and resilience have a role in milk variability and the current findings support and build on this concept. Likewise, Poppe et al., 2020 [16] suggested low variance of the deviations is thought to indicate good resilience, because resilient cows have a smaller range of deviations from their predicted lactation curve than less-resilient cows do. In this study, high variance was associated with a more negative emotional state (based upon the results of PC1). Whilst welfare (and emotional state) and resilience are two different concepts, the two are closely interlinked, as suggested by Colditz et al., 2016 [53]. They proposed that resilience can be described as the capacity of the animal to be minimally affected by a disturbance or to rapidly return to the physiological, behavioural, cognitive, health, affective and production states that pertained before exposure to a disturbance. The finding that cows with less milk variance are associated with more positive emotional states would suggest agreement with this, given that less milk variance in Poppe et al., 2020 [16] was associated with better resilience. Given milk-yield variability is often where production efficiency is lost as it suggests that the cows are not producing to optimum performance, being able to identify it may help to improve cattle welfare and performance. It is possible to hypothesise that recognising these cows and giving them more access to space and resources may improve their affective state and positive welfare assessment scores, as well as reducing their milk-yield variation, thereby increasing herd efficiency, particularly given that it has been shown that increased space leads to behaviour change and increased production [54].

Clustering cows based upon behaviour was shown to be possible in Craven et al., 2026 [23], and the concept has been extended here to cluster based upon QBA outcomes during the first 30DIM. The fact that these clusters were associated with differences in 100-day yield production and agree with the findings from the individual yield findings suggests that this concept may hold promise and should be studied further as if behaviour in early lactation could be shown to have predictive capabilities of a cow’s performance and welfare over the course of a lactation, then this would be significant for welfare assessment.

Changes in milk quality have also been studied at a herd level based upon affective states. Poppe et al., 2021 [55] reported low fat content as being associated with high variance and reduced resilience (amongst other parameters such as calving interval and survival to second lactation). In this study, fat was both associated with changes in principal component with increasing levels being associated with a more positive valence. This is conceptually in agreement with Poppe et al., 2021 [55] who showed that low fat content was associated with increased variability and therefore reduced resilience, assuming the link between welfare and resilience. The lack of significance for protein findings slightly contradict the herd-level findings in Popescu et al., 2025 who found higher PAI values, indicating more positive herd-level emotional states, were significantly associated with an increased fat-to-protein ratio (FPR) and a slightly lower protein content was observed, consistent with a dilution effect (due to higher yields). This should be explored further to see if it is related to herd-level effects.

At a herd level the association between increased milk yield and lower somatic cell count, and positive affective states (using a QBA-derived Positive Affect Index), has previously been reported [12], but this is the first study to use QBA to show the variation at an individual level within a herd. The fact that an elevated SCC is associated with a more negative emotional state, makes physiological sense. Mastitis is often sub-categorised into clinical and subclinical. Clinical mastitis is known to be painful and associated with inflammation and stress [56] but also agrees with des Roches et al., 2018 [18], de Boyer des Roches et al., 2017 [57] which used experimentally induced mastitis and QBA to show negative emotional states. Although often less considered, subclinical mastitis is also likely to cause discomfort and be painful given it reflects an inflammatory state. Peters et al., 2015 [58] found subclinical mastitis reduced thermal nociceptive threshold. In this study elevated SCC was used as an indicator of mastitis (given that cows with a SCC of greater than 200,000 are highly likely to be infected in at least one quarter [49]) and saw that increased SCC, using the binary threshold, was associated with more negatively associated terms. Pain has two components, sensory and affective, and it is likely that both will be reflected in a more negative behaviour as seen in the principal component scores. In the long term the effects of subclinical and clinical mastitis should be assessed, but the data did not allow this (both in terms of a limited number of observations (90) and lack of clinical history as the observer was only able to access milk recording data). However, this data was strengthened by the fact that it was truly blinded (the observer did not see the milk recording data or any health data until the end of the trial and data analysis commenced).

### Limitations

This study took place on one farm, over an extended period, with one scorer. This means that the generalisability of the findings is unknown, even though the findings appear to be biologically plausible and agree with the previous literature (although assessing slightly different metrics or with a different focus). Accuracy in the scoring, particularly over an extended period, was mitigated by using intra-observer reliability assessments (discussed further in Craven et al., 2026 [13]). The observer was also blinded to all the health and production information from the cows until the data analysis phase of the project, after the observations had ended, and therefore the observer was able to apply the QBA on the individual cows independently.

The schedule was designed as a compromise between frequency and feasibility given that regular assessment was shown to be important in early lactation [23]. The regular assessment at the end of the observation period was to ensure that if a sustained DIM effect was present that it could be detected later in lactation when otherwise the sampling was much less frequent. Although this is likely to have slightly affected the loadings in the PCA, the fact that Cow ID, date and PC score for the observation date were used in the analysis should have minimised downstream associations.

There was also a limitation that not all of the measurements were able to be taken from the office as it was hard to see and identify all of the cattle, for example when they were in cubicles. Although assessing the cattle at the cow level has been used in the literature for a standard observational study [25], rather than to test human–animal interactions and handling [59], it has not been used as a comparison between assessment at the cow level and at a distance. However, if cattle could not be identified from the office, assessment at the cow level was chosen as a compromise to prevent missing data points, and time to habituate was allowed for. If the observer felt that the cow was not showing natural behaviour, then the cow would not have been scored.

This study took place on a commercial farm and therefore there was potential for external factors to impact on QBA measurements, for example weather and changes in ration. Thus, the addition of date into the final models was utilised to reduce sampling uncertainty and account for shared temporal variation. Alongside the number of observations (*n* = 2700) and duration of study period, these effects were included to minimise potential noise in the data to help ensure associations of the studied variable. This is unlikely to apply for the milk-quality data which was performed on a much smaller dataset (*n* = 90) and therefore any associations can only be considered to be exploratory.

The data may also have been affected by confounding factors such as health, body condition score and social rank that were not controlled for but may have influenced both QBA and milk-production values.

QBA is often sometimes considered to be subjective and anthropomorphic, with concerns of judgement shift and bias, particularly given the longer time period (13 months) that this study was performed over. Given that the IOR showed high reliability with the observer, and the results largely agree or follow previous findings, it can be inferred that QBA outcomes described in this study play a role in inferring some concepts of emotional state assessment. Furthermore, significant associations with independently quantifiable variables over a long time period that the scorer was blinded to are worthy of consideration when assessing validity. Whilst QBA and milk production are far from the only potential welfare measurements, these outcomes can be used in conjunction with other methods to provide a more rounded measure of welfare within dairy cattle, and these results show an interesting area for further exploration as part of wider positive welfare assessment frameworks.

## 5. Conclusions

At an individual dairy-cow level, QBA scores indicative of more positive behavioural expression are associated with milk-production characteristics, particularly among first-lactation cows, and lower PC1 scores were associated with lower variability in daily milk production. Furthermore, the results suggested potential for behaviour in the first 30 days to have predictive effects on yield over the first 100 days in milk. Exploratory work suggested that QBA scores are also associated with changes in milk quality with the effects of milk fat and SCC being associated with changes in principal component. Increased fat follows the trends shown by yield for first-lactation cows and increase with a more positive emotional state, whereas an SCC associated with >200,000 cells/ml (used as an indicator of increased likelihood of intramammary infection) is associated with a more negative state.

## Figures and Tables

**Table 1:** Stable loadings for PC1 (19) and PC2 (17) with their 95% confidence intervals. The factors are listed in order of magnitude of loading. The dataset included 2700 observations, across 123 dates, and included 114 individual cows. Only stable loadings were included.

Variable	Component	Loading	CI Low	CI High	Stable
Calm	PC1	−0.35	−0.36	−0.35	TRUE
Content	PC1	−0.35	−0.36	−0.35	TRUE
Relaxed	PC1	−0.35	−0.36	−0.35	TRUE
Happy	PC1	−0.35	−0.35	−0.34	TRUE
Uneasy	PC1	0.33	0.32	0.33	TRUE
Positively occupied	PC1	−0.32	−0.33	−0.32	TRUE
Fearful	PC1	0.30	0.30	0.31	TRUE
Agitated	PC1	0.21	0.20	0.22	TRUE
Distressed	PC1	0.17	0.14	0.19	TRUE
Sociable	PC1	−0.16	−0.18	−0.15	TRUE
Friendly	PC1	−0.15	−0.17	−0.13	TRUE
Apathetic	PC1	0.15	0.13	0.17	TRUE
Lively	PC1	−0.13	−0.15	−0.11	TRUE
Irritable	PC1	0.12	0.10	0.14	TRUE
Indifferent	PC1	0.09	0.07	0.11	TRUE
Frustrated	PC1	0.08	0.07	0.11	TRUE
Active	PC1	0.08	0.05	0.10	TRUE
Bored	PC1	0.03	0.02	0.05	TRUE
Playful	PC1	0.03	0.02	0.06	TRUE
Active	PC2	0.48	0.36	0.53	TRUE
Playful	PC2	0.39	0.28	0.47	TRUE
Inquisitive	PC2	0.33	0.25	0.38	TRUE
Lively	PC2	0.33	0.20	0.39	TRUE
Frustrated	PC2	0.29	0.23	0.40	TRUE
Bored	PC2	0.27	0.09	0.41	TRUE
Friendly	PC2	0.24	0.11	0.34	TRUE
Distressed	PC2	0.21	0.15	0.29	TRUE
Sociable	PC2	0.18	0.08	0.26	TRUE
Agitated	PC2	0.18	0.09	0.27	TRUE
Irritable	PC2	0.14	0.08	0.20	TRUE
Fearful	PC2	−0.12	−0.17	−0.07	TRUE
Positively occupied	PC2	−0.11	−0.14	−0.07	TRUE
Uneasy	PC2	−0.06	−0.10	−0.02	TRUE
Content	PC2	0.05	0.03	0.09	TRUE
Happy	PC2	0.05	0.03	0.08	TRUE
Calm	PC2	0.04	0.01	0.09	TRUE

**Table 2:** (**A**) Fixed-effect estimates from the final linear mixed-effects model examining associations between total daily milk yield and behavioural dimensions (PC1 and PC2), lactation number, and days in milk (DIM). Lactation 1 was used as the reference category. The dataset included 2700 observations from 114 cows across 123 assessment dates; (**B**) estimated associations between PC1 and PC2 and total milk yield by lactation; (**C**) estimated associations between PC1 and PC2 and total milk yield between lactation.

(**A**)					
	**Estimate**		**Standard Error**	**95% CI**	***p*-Value**
Intercept	15.338		1.354	12.685–17.992	
PC1	−0.241		0.100	−0.438–−0.045	0.016 *
PC2	0.146		0.172	−0.191–0.483	0.397
Lactation 1	(Ref)				
Lactation 2	7.355		1.629	4.161–10.548	<0.001 ***
Lactation 3+	1.963		1.704	−1.377–5.303	0.249
PC1: Lactation 2	0.194		0.131	−0.063–0.451	0.139
PC1: Lactation 3+	0.388		0.139	0.116–0.661	0.005 **
PC2: Lactation 2	−0.099		0.209	−0.508–0.311	0.637
PC2: Lactation 3+	0.213		0.262	−0.300–0.725	0.417
DIM1 (spline)	18.180		0.861	16.492–19.867	<0.001 ***
DIM2 (spline)	15.726		1.285	13.207–18.238	<0.001 ***
DIM3 (spline)	4.159		1.304	1.604–6.714	0.001 **
DIM4 (spline)	17.515		1.461	14.651–20.379	<0.001 ***
DIM5 (spline)	−1.515		1.042	−3.558–0.528	0.146
(**B**)					
**Principal Component**	**Lactation**	**Slope**	**Standard Error**	**95% CI**	***p*** **-Value**
PC1	1	−0.241	0.100	−0.438–−0.045	0.016 *
	2	−0.047	0.093	−0.229–0.134	0.610
	3	0.147	0.103	−0.054–0.349	0.152
PC2	1	0.146	0.172	−0.191–0.483	0.397
	2	0.047	0.119	−0.186–0.280	0.693
	3	0.358	0.198	−0.030–0.746	0.070
(**C**)					
**Principal Component**	**Lactation**		**Estimate**	**Standard Error**	***p*** **-Value**
PC1	Lactation 1–Lactation 2		−0.194	0.131	0.302
	Lactation 1–Lactation 3		−0.389	0.139	0.014 *
	Lactation 2–Lactation 3		−0.195	0.133	0.310
PC2	Lactation 1–Lactation 2		0.099	0.209	0.885
	Lactation 1–Lactation 3		−0.213	0.262	0.695
	Lactation 2–Lactation 3		−0.311	0.230	0.366

*** *p* < 0.001, ** *p* < 0.01, * *p* < 0.05.

**Table 3:** (**A**) Fixed-effect estimates from the final linear mixed-effects model examining associations between log milk-yield variation and behavioural dimensions (PC1 and PC2) and lactation number. Lactation 1 was used as the reference category. The dataset included 1607 observations from 88 cows across 94 assessment dates; (**B**) associations between principal components and LnVar with sensitivity analysis accounting for temporal autocorrelation. Primary model: linear mixed-effects model including PC1, PC2 and lactation as fixed effects, with random intercepts for Cow ID and date. CAR(1) sensitivity model: continuous-time AR(1) correlation structure within Cow ID. The estimated within-cow correlation parameter was 0.847. The CAR(1) model did not include the date random effect: it was used specifically to assess the robustness of the associations to temporal autocorrelation.

(**A**)						
	**Percentage Change in Variability**		**95% CI**	***p*-Value**		
Intercept	1109.23		915.46–1339.96			
PC1	3.63		1.14–6.17	0.004 **		
PC2	−1.43		−5.02–2.30	0.447		
Lactation 1	(Ref)					
Lactation 2	38.66		9.39–75.76	0.007 **		
Lactation 3+	20.98		−6.22–56.06	0.141		
(**B**)						
	**Primary Model**			**CAR(1) Sensitivity Model**		
**Predictor**	**Estimate**	**95% CI**	***p*-Value**	**Estimate**	**95% CI**	***p*-Value**
PC1	0.0356	0.0114–0.0598	0.004	0.0310	0.0078–0.0541	0.009
PC2	−0.0144	−0.0515–0.0227	0.447	−0.0155	−0.0502–0.0191	0.379
Lactation 2	0.3269	0.898–0.5639	0.007	0.3089	0.0711–0.5468	0.012
Lactation 3+	0.1905	−0.0642–0.4451	0.141	0.1798	−0.0759–0.4356	0.166

** *p* < 0.01.

**Table 4:** Fixed-effect estimates from the final linear mixed-effects model examining associations between 100-day milk yield and behavioural clusters for PC1 and PC2. Cluster 1 was used as the reference category. The dataset included 93 cows (3 cows were excluded from the analysis as although they were allocated to a cluster; they exited the trial before 100 days in milk). All 3 cows were in cluster 2 for PC1 and cluster 1 for PC2.

Milk Yield	Clusters	Estimate	SE	95% CI	*p*
100 days	Intercept	2696.8	226.3	2247.2–3146.4	
	PC1 Cluster 1	(Ref)			
	PC1 Cluster 2	529.4	225.8	80.8–978.0	0.021 *
	PC2 Cluster 1	(Ref)			
	PC2 Cluster 2	1158.5	282.0	598.2–1718.8	<0.001 ***
	PC2 Cluster 3	552.6	219.8	115.8–989.4	0.014 *

*** *p* < 0.001, * *p* < 0.05.

**Table 5:** Fixed-effect estimates from the final linear mixed-effects model examining associations between milk fat (kg) and behavioural dimensions (PC1 and PC2), lactation number, total daily yield and DIM. Lactation 1 was used as the reference category. The dataset included 90 observations from 74 cows across 10 assessment dates.

Model Outcome	Explanatory Variables	Coefficient	Standard Error	95% CI	*p*-Value
Total fat (kg)	Intercept	0.2771	0.1016	0.0749–0.4793	
	PC1	−0.0265	0.0179	−0.0467–−0.0063	0.0117 *
	PC2	−0.0308	0.0179	−0.0676–0.0061	0.0976
	Lactation 1	(Ref)			
	Lactation 2	−0.2073	0.0823	−0.3715–−0.0431	0.0141 *
	Lactation 3+	−0.1641	0.0829	−0.3294–0.0012	0.0516
	Total daily yield	0.0386	0.0029	0.0328–0.0445	<0.001 ***
	DIM	−0.0008	0.0004	−0.0015–0.00003	0.040 *

*** *p* < 0.001, * *p* <0.05.

**Table 6:** Odds ratios (OR) and 95% CI from a binomial logistic regression model with standard errors adjusted for clustering by cow, associated with a positive SCC outcome. QBA before milk recording (one day date differential) and lactation 1 were used as reference categories. The dataset included 90 observations from 74 cows across 10 assessment dates.

Model Outcome	Explanatory Variables	Odds Ratio	95% CI	*p*-Value
SCC (binary)	Intercept	0.16	0.03–0.97	
	PC1	1.67	1.03–2.69	0.036 *
	PC2	1.47	0.87–2.49	0.148
	−1 SCC differential	(Ref)		
	Zero date differential (milk recording and QBA same day)	0.83	0.13–5.18	0.843
	One day differential (QBA after milk recording)	1.25	0.43–3.63	0.677
	Lactation 1	(Ref)		
	Lactation 2	0.67	0.12–3.77	0.650
	Lactation 3+	0.58	0.12–2.78	0.495
	DIM	1.01	1.00–1.02	0.187

* *p* <0.05.

## Data Availability

The raw data supporting the conclusions of this article will be made available by the authors on request.
